# The impact of the Iranian health transformation plan policy on equitable access to medical imaging services in West Iran

**DOI:** 10.1186/s13104-023-06634-2

**Published:** 2023-11-27

**Authors:** Maryam Saran, Banafsheh Darvishi Teli, Aziz Rezapour, Soraya Nouraei Motlagh, Meysam Behzadifar, Payam Haghighatfard, Nicola Luigi Bragazzi, Masoud Behzadifar

**Affiliations:** 1 Social Determinants of Health Research Center, Lorestan University of Medical Sciences, Khorramabad, Iran; 2 Health Management and Economics Research Center, Iran University of Medical Sciences, Tehran, Iran; 3 Human Nutrition Unit Department of Food and Drugs, University of Parma Medical School, Building C, Via Volturno, 39, Parma, 43125 Italy

**Keywords:** Diagnostic imaging, Equity in Healthcare, Gini Coefficient, Health Economics, Health Policy, Health Transformation Plan, Iran, Lorenz Curve

## Abstract

**Objective:**

Equity in the delivery of health services, including diagnostic imaging, is crucial to achieving universal health coverage. The Health Transformation Plan (HTP), launched in 2014, represents a major healthcare policy to improve the quality and accessibility of healthcare services. This study aimed to explore the impact of the HTP on equity in the access to medical imaging in Lorestan province, located in west Iran, from 2014 to 2023. Annual growth rates (AGR) of imaging devices were calculated, whilst equity assessment of medical imaging distribution was carried out by means of the Gini coefficient and the Lorenz curve per 100,000 population. The latter was generated using the cumulative distribution of imaging devices, as well as the cumulative population ratio.

**Results:**

Between 2014 and 2023, the number of imaging devices has increased threefold. The AGR of installing CT and MRI scanners in Lorestan province increased between 2014 and 2023. The Gini coefficients increased from 0.12 for CT and 0.16 for MRI in 2014 to 0.33 in 2023 for both devices. This indicates a decrease in equity in access to these fundamental health technologies despite the increase in their figures. Policymakers should better allocate medical equipment based on the specific health needs of different regions throughout Iran.

**Supplementary Information:**

The online version contains supplementary material available at 10.1186/s13104-023-06634-2.

## Introduction

Equity is a fundamental principle in achieving universal health coverage (UHC), which means that everyone, regardless of their socio-economic status, geographic location, or other factors, has access to quality health services without suffering from financial hardship [1]. Achieving equity in the delivery of health services and provisions is crucial to achieving UHC because it helps ensure that everyone has access to the health services they need, regardless of their ability to pay [2]. Governments and health systems must prioritize equity in the access to health services by allocating resources to areas with the greatest need, implementing policies and programs that target vulnerable populations, and improving the quality of health services in underserved areas [3].

Computed tomography (CT) and magnetic resonance imaging (MRI) are both imaging techniques that are commonly used in the diagnosis and prognosis of diseases [4]. They play important roles in identifying and characterizing a wide range of medical conditions [5]. When used appropriately, these imaging techniques can provide valuable diagnostic information to help healthcare providers make accurate diagnoses and develop effective treatment plans for their patients [6].

The Health Transformation Plan (HTP) is a major healthcare policy implemented by the government of Iran in 2014. The main objective of the HTP was to improve the quality and accessibility of healthcare services, especially for vulnerable populations [7]. The HTP includes plans to build new hospitals and health centers, as well as to upgrade existing facilities with modern equipment, infrastructure, and technologies [8]. Several studies have examined the effects of Iran’s HTP on healthcare performance, quality, and related indicators [9–11]. The present study aims to explore the impact of the HTP on equity in the access to medical imaging (CT and MRI scanners) in Lorestan province from 2014 to 2023, providing insights into the local effects of a nationwide policy, as a model for similar investigations worldwide. The findings aim to inform resource allocation and healthcare infrastructure development, ensuring fair distribution of advanced diagnostics and supporting the goals of healthcare policies like the HTP.

## Main text

### Methods

#### Data collection

Lorestan province is located in western Iran and comprises 11 cities with a total population of approximately 1.8 million people. The Lorestan University of Medical Sciences (LUMS) is responsible for ensuring the health and well-being of the public through the provision of essential services. In this province, there are 14 public hospitals in its 9 cities. Population figures for each city in 2014 and 2023 were obtained from the Statistical Center of Iran (at https://www.amar.org.ir). Similarly, information regarding the number of CT and MRI scanner**s** in 2014 and 2023 was obtained from the Vice-Chancellor of LUMS, at https://www.lums.ac.ir.

#### Annual growth rates (AGRs)

The annual growth rates (AGRs) of the two types of medical imaging (CT and MRI scanners) were also calculated from 2014 to 2023. The formula of AGR is reported in the supplementary material (Appendix 1).

#### Equity assessment

We employed the Gini coefficient and the Lorenz curve to assess the distribution equality of CT and MRI scanners across the various cities in Lorestan province [5]. The Lorenz curve was generated using the cumulative distribution of CT and MRI scanners, as well as the cumulative population ratio, which is a method commonly used in public health economics [12, 13]. In general, the closer the Lorenz curve is to the line of equality, the more equitable the distribution of services [14]. The Gini coefficient ranges from 0 to 1, with values closer to 0 indicating greater equality in distribution and higher values indicating greater inequality [14]. The formula of the Gini coefficient is in the supplementary material (Appendix 2).

### Statistical analysis

The R software Version 3.6.1 via “DescTools” package was used for all statistical analyses. *P*-value < 0.05 was considered significant. Descriptive analysis was used to compute the numbers of CT-scans and MRIs in terms of absolute figures and per 100,000 population in the study cities.

## Results

Table 1 shows the distribution of CT and MRI scanners devices in public hospitals across each city in 2014 and 2023.

**Table 1:** Number of CT and MRI scanners per 100,000 populations in the study cities

City	Number of Public hospitals	2014					2023				
		Population	Number of CT scanners	Number of examinations	Number of MRI scanners	Number of examinations	Population	Number of CT scanners	Number of examinations	Number of MRI scanners	Number of examinations
Azna	1	65,613	1	5167	0	0	74,936	1	8032	0	0
Aligoudarz	2	123,127	1	3962	0	0	137,534	2	7019	1	0
Boroujerd	2	287,214	1	6831	1	812	326,452	2	19,627	1	612
Delfan	1	132,291	0	0	0	0	143,973	1	4618	1	0
Doroud	1	162,841	0	0	0	0	174,508	1	2917	0	0
Poldokhatr	1	68,714	0	0	0	0	73,744	1	4186	1	0
Khorramabd	3	483,613	2	69,173	1	2692	506,471	5	114,083	1	6193
Kohdasht	1	143,612	0	0	0	0	166,658	1	4816	1	0
Selseleh	1	69,301	0	0	0	0	75,559	1	3018	0	0

**Fig. 1:**
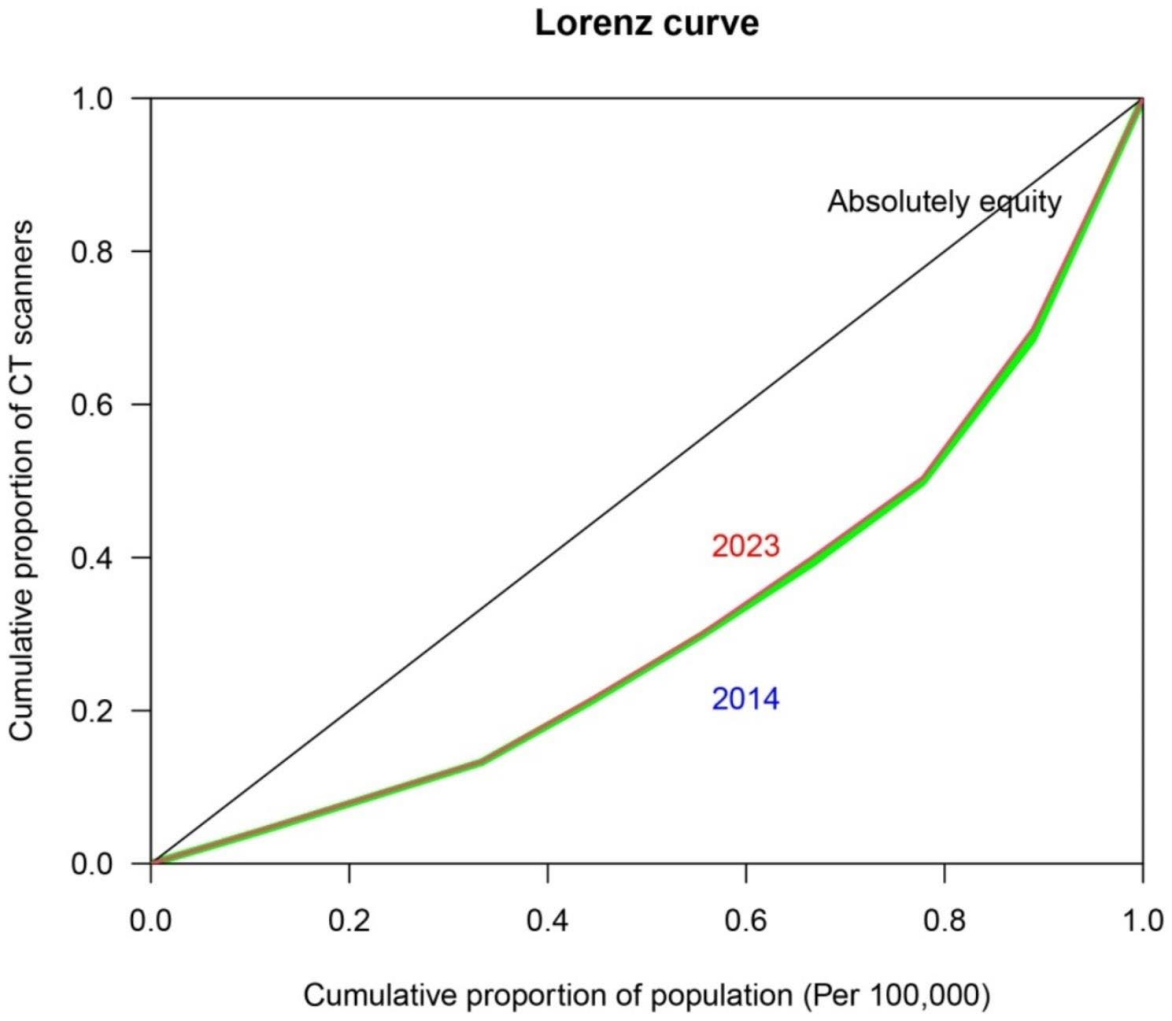
Lorenz curve evaluating the fairness in access to CT scanners in Lorestan province, west Iran, from 2014 to 2023

Between 2014 and 2023, the number of CT and MRI scanners has increased threefold. However, between 2014 and 2023, the growth of CT scanners was higher than that of MRI scanners. The cities of Khorram Abad, Borujerd, and Aligoudarz exhibited the highest growth in these devices. The Gini coefficient of CT scanners was 0.16 in 2014 and increased to 0.33 in 2023, indicating a decrease in equity in access to this health technology despite the increase in the number of CT scanners (Fig. 1).

A Gini coefficient of 0 represents perfect equity, indicating that everyone has equal access to the resource in question. In contrast, a Gini coefficient of 1 signifies complete inequality, where a few have all the resources and others have none. Therefore, a Gini coefficient of 0.16 in 2014 suggests a relatively low level of inequality in access to CT-scans in Lorestan province. However, the increase to 0.33 in 2023 indicates a substantial increase in inequality over the years. There is no universally acknowledged or fixed threshold for what constitutes an equitable or inequitable Gini coefficient because it can vary depending on the context. In the case of medical imaging technology, a higher Gini coefficient indicates more unequal access. So, the estimated AGR for CT scans from 2014 to 2023 was approximately 10.66%.

**Fig. 2:**
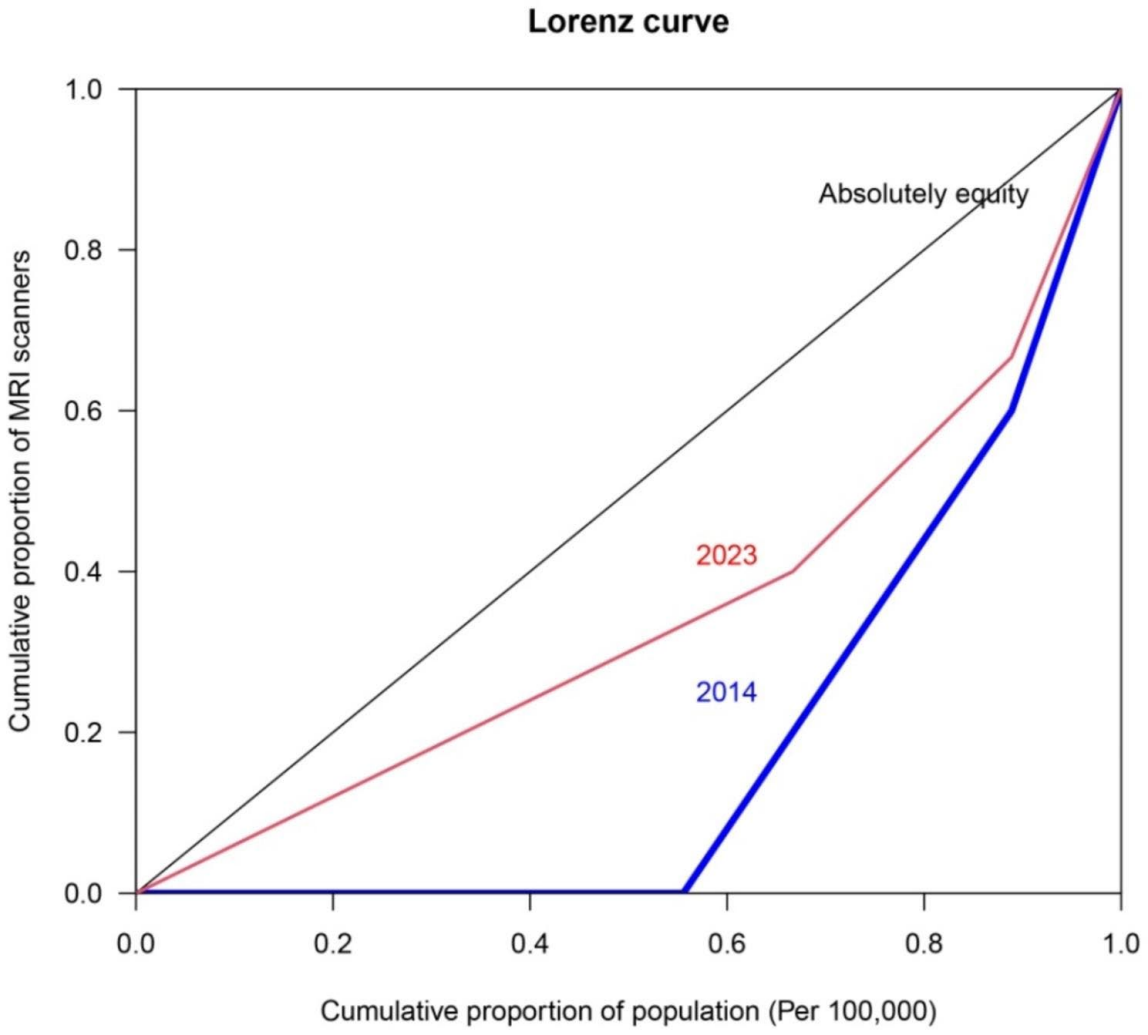
Lorenz curve evaluating the fairness in access to MRI scanners in Lorestan province, west Iran, from 2014 to 2023

Also, the Gini index in evaluating the fairness of the use of MRIs during the mentioned period indicated the worsening of the equity distribution of this device in the population of Lorestan province, so that the Gini index increased from 0.12 to 0.33 during the years 2014 to 2023 (Fig. 2).

This change in the Gini coefficient signifies a noticeable shift in the distribution of access to MRIs in Lorestan province over the decade. The increase from 0.12 to 0.33 suggests a substantial rise in inequality during this period. The estimated AGR for MRI from 2014 to 2023 was approximately 6.35%.

The AGR for CT-scans stood at approximately 10.66%, suggesting a consistent upward trend in their utilization. This rise may reflect an increased demand for CT-scans or improved accessibility to these diagnostic services, potentially signaling enhancements in healthcare delivery. Meanwhile, MRIs exhibited an AGR of approximately 6.35%, underscoring an ongoing favorable trend in their usage. This growth could be attributed to advancements in healthcare practices, technological innovations, or evolving medical requirements within the region.

## Discussion

The findings of our study showed that the number of CT and MRI scanners increased between 2014 and 2023 with the start of the HTP. The government’s HTP allocated a significant amount of economic-financial resources into the healthcare sector. Many government hospitals acquired modern equipment to provide superior services to patients. In the reforms of the health system in Turkey, one of the goals was to eliminate inequalities in financing and access to health services through the provision of special equipment and services. The Turkish government has allocated effective financial resources for the increase of disease diagnostic devices [15]. Similarly, with the increase of CT and MRI scanners in Lorestan province, we have seen an increase in these services between 2014 and 2023 in government hospitals. In 2009, new reforms in the health system began in China which increased people’s access to diagnostic services [16]. The findings of the studies conducted in Turkey and China showed that the allocation of appropriate financial resources has increased diagnostic devices and services provided in the health sector, which is consistent with our findings [15, 17].

Our research revealed that larger and more developed cities in the province had a higher distribution of CT and MRI scanners. This can be attributed to the greater number of public hospitals and medical professionals in these cities, who require more medical equipment [18]. In smaller cities, the patient volume is lower, and the government policy is to develop and equip larger cities, which have more specialist doctors and specialized hospitals. Public hospitals in Iran operate with the profits from their activities and government budgets, and the use of diagnostic devices must be economically justified [19].

In Lorestan province, in different cities, the number of hospital beds, specialist doctors, outpatient and inpatient visits can affect the distribution and usage of diagnostic imaging devices. In a study, the distribution of CT and MRI scanners in 66 cities in China was investigated [5]. In this study, the AGR increased and the number of allocated of CT and MRI scanners correlated with variables such as population of the region, GDP, number of hospitals, hospital beds, outpatient and inpatient visits and health professionals which was consistent with the findings of our study. One of the reasons can be that, in Iran, public hospitals rely on their activities’ profits and government budgets, so the use of diagnostic devices should be economically justified. In various studies conducted in other countries, findings show that due to the costs of installing and maintaining diagnostic imaging devices, health systems give priority to cities that have physicians and more requests to use these services. in line with our findings [20, 21].

Despite the increase of CT and MRI scanners between 2013 and 2014 in Lorestan province, witnessing an increase in the Gini coefficients, it can be said that there has been a decrease in equity in access to diagnostic imaging services. In the studies conducted in China [5], Japan [20] and Myanmar [21], the findings show that with the increase of CT and MRI scanners between different years, in addition to the annual growth, there has been a decrease in the Gini coefficient and an increase in equity in access to these services, which is different from the findings of our study. Appropriate geographical distribution of doctors, attention to population, number of hospital beds, proper prioritization of resources, decision-making based on scientific evidence and the existence of teaching hospitals were among the reasons for reducing the Gini coefficient.

Some cities in Lorestan province lack the demand for CT and MRI scanners, yet they were equipped with these devices due to political influence and lobbying. Also, social media and local media have created expectations for luxury diagnostic devices, adding to the asymmetry of information in the health system.

### Policy recommendation

When procuring and installing CT and MRI scanners, it is imperative to take into account the projected population coverage and their anticipated future usage. To further alleviate the burden on public healthcare institutions and promote equitable access to imaging services, harnessing the private sector’s capacity for device installation and service provision is essential. This can be achieved through the establishment of appropriate insurance support mechanisms, fostering collaboration between the public and private sectors to enhance access and the quality of care. Such a comprehensive approach can lead to more efficient healthcare resource allocation and better healthcare services for the Iranian population.

In conclusion reasonable allocation and resource use should be based on prioritization variables such as population density and device demand for them, and the allocation of resources in the health sector should be based on need and political priority should not be taken into account.

### Limitations

One limitation of this study was the lack of suitable data for analyzing the impact of increasing CT and MRI scanners on clinical activities in public hospitals of Lorestan province. It should also be noted that the Gini coefficient is only interpretable in a relative sense, and there is no clear definition of what constitutes a low or high value. Another limitation was the lack of access to data of different Iranian provinces that hinders a broader picture of the impact of the HPT on the distribution of CT and MRI scanners.

### Electronic supplementary material

Below is the link to the electronic supplementary material.

Supplementary Material 1

## Data Availability

The datasets used and/or analysed during the current study are available from the corresponding author on reasonable request.

## Abbreviations

UHC: Universal health coverage
CT: Computed tomography
MRI: Magnetic resonance imaging
HTP: Health transformation plan
AGRs: Annual growth rates
OOP: Out-of-pocket
HTA: Health technology assessment
