# Supplementary material for: The impact of the Iranian health transformation plan policy on equitable access to medical imaging services in West Iran
Source: BMC Res Notes. 2023 Nov 27;16:350. doi: 10.1186/s13104-023-06634-2 (PMC10680178; doi:10.1186/s13104-023-06634-2)
Supplement: Supplementary file 1 — Supplementary Material 1 [file 13104_2023_6634_MOESM1_ESM.docx]

**Appendix 1: The formula of annual growth rates (AGRs):**

### AGR=$\sqrt[\boldsymbol{N}]{\frac{\boldsymbol{X}\boldsymbol{2}}{\boldsymbol{X}\boldsymbol{1}}}$ – 1: (X2: quantity of CT-Scans or MRIs scanners in 2023, X1: quantity of CT-scans or MRIs scanners in 2014, N: number of years).

**Appendix 2: The formula of the Gini coefficient is:**

G=$\sum_{i=1}^{n} P_{i}Y_{i}+2\sum_{i=1}^{n-1} P_{i}(1-V_{i})-1$,

for our study $P_{i}$is the cumulative share of the population in each city;

$Y_{i}:$is the cumulative share of health technology in each city; and

$V_{i}$ : in terms of health resource priority-setting, is the fractional rank (from the lowest to the highest).
